# Modeling of inter-organizational coordination dynamics in resilience planning of infrastructure systems: A multilayer network simulation framework

**DOI:** 10.1371/journal.pone.0224522

**Published:** 2019-11-13

**Authors:** Qingchun Li, Shangjia Dong, Ali Mostafavi

**Affiliations:** Zachry Department of Civil and Environmental Engineering, Urban Resilience, Networks, and Informatics Lab, Texas A&M University, College Station, Texas, United States of America; Universitat de Barcelona, SPAIN

## Abstract

This paper proposes and tests a multilayer framework for simulating the network dynamics of inter-organizational coordination among interdependent infrastructure systems (IISs) in resilience planning. Inter-organizational coordination among IISs (such as transportation, flood control, and emergency management) would greatly affect the effectiveness of resilience planning. Hence, it is important to examine and understand the dynamics of coordination in networks of organizations within and across various systems in resilience planning. To capture the dynamic nature of coordination frequency and the heterogeneity of organizations, this paper proposes a multilayer network simulation framework enabling the characterization of inter-organizational coordination dynamics within and across IISs. In the proposed framework, coordination probabilities are utilized to approximate the varying levels of collaboration among organizations. Based on these derived collaborations, the simulation process perturbs intra-layer or inter-layer links and unveils the level of inter-organizational coordination within and across IISs. To test the proposed framework, the study examined a multilayer collaboration network of 35 organizations from five infrastructure systems within Harris County, Texas, based on the data gathered from a survey in the aftermath of Hurricane Harvey. The results indicate that prior to Hurricane Harvey: (1) coordination among organizations across different infrastructure systems is less than the coordination within the individual systems; (2) organizations from the community development system had a low level of coordination for hazard mitigation with organizations in flood control and transportation systems; (3) achieving a greater level of coordination among organizations across infrastructure systems is more difficult and would require a greater frequency of interaction (compared to within-system coordination). The results show the capability of the proposed multilayer network simulation framework to examine inter-organizational coordination dynamics at the system level (e.g., within and across IISs). The assessment of inter-organizational coordination within and across IISs sheds light on important organizational interdependencies in IISs and leads to recommendations for improving the resilience planning process.

## Introduction

Natural hazards (e.g., hurricanes, sea-level rise, earthquakes, and flooding) pose great threats to infrastructure systems that support the well-being of society. Resilience, the “ability to prepare and plan for, absorb, recover from or more successfully adapt to actual or potential adverse events” is regarded as an important capacity of a city or a community facing stressors [1,2]. Hence, resilience planning that integrates hazard mitigation across interdependent infrastructure systems (IISs) and proactively deals with urban system hazards is an essential element in successful hazard mitigation implementation [3,4].

Resilience planning involves coordination of multi-organizational processes across IISs [5]. Contradictions and inconsistencies between plans would be expected if the level of coordination among diverse organizations is insufficient [6]. Organizations of different infrastructure systems usually have different priorities and preferences pertaining to development, hazard mitigation, and resilience improvement [7,8]. For example, in the case of resilience planning for flooding, it is common for organizations from transportation systems to be more concerned about infrastructure development to solve traffic congestion, while organizations of flood control entities and environment conservation groups focus more on hazard mitigation and environment preservation. In addition, the level of coordination across various infrastructure systems may understandably be lower than the level of coordination within the same system. Hence, to get better resilience planning, it is essential to examine and understand coordination dynamics among organizations within and across IISs.

The planning background in Houston also highlights the necessity of examining and understanding inter-organizational coordination dynamics within and across IISs for better resilience planning. Houston is one of the most flood-prone city in the nation. One important reason contributing to flood vulnerability in Houston is the confliction between the rapid urban development and poor urban planning with underinvestment in flood control infrastructure systems [1]. Houston is the only city without zoning policies in North America and is well known for its modest land use regulations [9]. Growing urbanism (leading to more dense development patterns) in Houston has made the city vulnerable to natural hazards due to incompatible investment on hazard mitigation infrastructure [1]. Insufficient integration of land use approaches and hazard mitigation strategies with infrastructure plans and projects has increased both social and physical vulnerability [3]. The insufficient integration among hazard mitigation, land use, and infrastructure plans is, to some extent, due to inadequate coordination for resilience planning of IISs across different infrastructure systems, such as the flood control and transportation system [4,10–12].

For example, this insufficient integration among plans (due to inadequate coordination among actors) was problematic and led to vulnerability during Hurricane Harvey. In August 2017, Hurricane Harvey hit Houston Texas, and caused an estimated $125 billion in damage [13]. One of the important reasons that Hurricane Harvey inflicted huge losses in the Houston area was the release of two flood control reservoirs (i.e., the Barker and Addicks reservoirs, built in the 1940s). The flood water released to downstream neighborhoods in West Houston caused inundation of more than 9,000 houses for more than two weeks. The West Houston area has never flooded before and did not even flood before the release during Hurricane Harvey. The decision to release flood water was mainly to protect the reservoirs from breaching and preventing even more catastrophic losses. The high-water level in the reservoirs was not only due to the unprecedented rainfall by Hurricane Harvey, but also because of infrastructure development close to the reservoir areas surrounding the newly constructed segment of State Highway 99 (SH-99). While constructing the SH-99 segment intended to improve the roadway network and alleviate the traffic burden in Houston, the inconsistent transportation plans and flood control plans allowed increased development around reservoirs. Such development increased paved area by eliminating the wetlands that could store and absorb the water without increasing the burden of the reservoirs. The example highlights the interdependencies among IISs and negative effects of inadequate cross-system coordination for hazard mitigation in resilience planning.

Interdependencies among IISs is an important aspect of coordination in the resilience planning process. While several studies have examined the interdependencies among IISs, the majority of the existing literature primarily focuses on physical aspects [14–16], and little is known regarding the dynamics of inter-organizational coordination. Coordination among organizations can be conceptualized graphically as the links between nodes in network theory [17,18]. In other words, networks are structures upon which the coordination behavior of IISs involved in resilience planning unfolds. Hence, analyzing the structure and characteristics of inter-organizational networks can provide important insights regarding the dynamics of coordination in resilience planning of IISs. In one stream of research, various studies adopted network analysis in assessing the properties of social networks involved in hazard mitigation, resilience planning, and emergency response. Kapucu [17] studied the dynamics of inter-organizational networks in response to the terrorist attacks on September 11, 2001. Bodin and Crona [19] discussed how the characteristics of social networks (e.g., density, centrality, core-periphery, and level of cohesion) affect natural resource governance for the resilience of social-ecological systems. Magsino [20] concluded the applications of social network analysis (SNA) for building community resilience to disasters. Mills et al. [21] adopted SNA to understand roles of stakeholders in the systematic planning process, linking regional planning to location actions. Most of the extant works of literature adopted SNA to gather important information regarding the structure and node properties (such as the importance of organizations and their centrality) of inter-organizational networks.

While SNA informs about the structural properties of inter-organizational networks [22,23], there are multiple factors need to be considered when examining coordination dynamics in resilience planning of IISs using SNA. First, in resilience planning coordination, a link between two organizations represents their communication and interaction, and this can have varying levels of frequency. For example, organizations A and B might collaborate once a year or once a week, and intuitively, a greater frequency of collaboration means more coordination among organizations. Hence, to fully capture the dynamics of coordination, an appropriate network analysis should be able to capture and simulate the varying levels of interaction frequencies among organizations. Second, a proper network analysis needs to consider inter-organizational coordination within and across different infrastructure systems. Therefore, the analysis should enable evaluating interactions among nodes of different types. This aspect is particularly important in examining coordination among organizations within and across IISs, because in resilience planning, the coordination among organizations within the same system (e.g., organizations in transportation systems) might have different frequency compared to coordination among organizations across different systems (e.g., organizations in transportation and flood control systems). Hence, an appropriate methodology should enable representing organizations from different infrastructure systems in a separate layer and facilitate a multi-layer modeling of inter-organizational coordination networks.

Considering these factors, this study proposes a network simulation process to capture the varying levels of interaction frequencies among organizations and employs a multilayer network approach to representing the coordination between organizations of IISs. We convert the collaboration at varying levels of frequency among organizations to the link probability in the network representing the likelihood that collaboration may happen among organizations. Accordingly, perturbations in the links of the network (based on their coordination probabilities) are used to simulate the dynamics of inter-organizational coordination during a time period (e.g., one year). The changes in network-level measures after link perturbation is then used to evaluate coordination performance. The existing literature has specified the common procedure for network characterization based on evaluating network performance: (1) obtaining empirical data to map a network; (2) measuring the investigated network’s features; (3) conducting link or node perturbation in the network [24]; (4) assessing the network performance after perturbation [24,25]. For example, Albert et al. randomly removed a small number of nodes in a network and evaluated the network performance to study the network resilience to failure, finding that a scale-free network has both error tolerance and targeted attack vulnerability properties [26]. Larocca used node perturbation to simulate random failures in an electric power system caused by operator errors and aging components to evaluate robustness of the electric power system [27]. The results indicated the capability of the simulation model to estimate actual performance of the electric power system. Dong et al. studied transportation network resilience by node and link removal approaches to simulate the network disruption effect [28–31]. The results of the research helped to identify the vulnerability of transportation networks to natural disasters.

**Fig 1:**
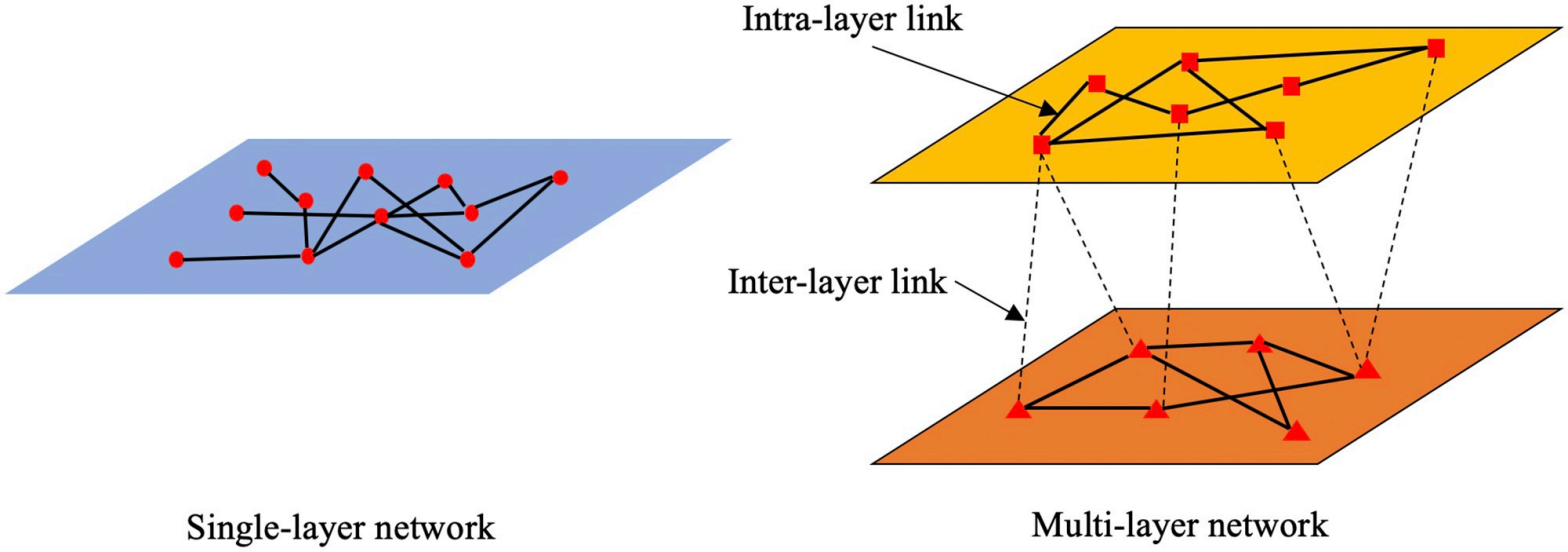
Single-layer network and multi-layer network.

The majority of studies regarding infrastructure systems employing network modeling focused primarily on physical interdependencies among IISs. In addition, most of these studies did not fully consider the interactions among organizations managing and operating these systems (e.g., inter-organizational coordination in resilience planning process among IISs) [25,28,31,32]. In this study, we adopt a multi-layer network analysis framework to represent the interactions among organizations of IISs. Multilayer networks enable studying networks with different types of connections, a ubiquitous characteristic in social and engineering systems [33]. Fig 1 illustrates a schematic representation of the single-layer network and multi-layer network. Extensive research has been conducted on interdependency analysis within urban systems using multilayer network tools. For example, Cardillo et al. studied the resilience of the air transportation network using a multiplex network formalism [34]; Zhu and Mostafavi investigated critical organizations in the disaster response system by the meta-network representing different types of entities in disaster response systems (e.g., organizations, tasks, information and resources) [22]; Fan and Mostafavi studied disaster management system-of-systems (DM-SoS) by establishing the meta-network framework including stakeholder, information, resource, operation, and policy networks [23]; and Solé-Ribalta et al. studied congestion in transportation networks using multiple layers to represent short range transportation and long range transportation [35]. While multi-layer-network analysis has been utilized in the analysis of interdependencies among systems and processes of IISs, its application is rather limited in examining inter-organizational coordination for resilience planning in IISs. The multilayer network provides a novel approach to studying the inter-organizational coordination within and across IISs. In this approach, organizations are grouped by different infrastructure systems (such as transportation, flood control, and emergency response) to study inter-organizational coordination for hazard mitigation within and across IISs affecting resilience planning [5]. Hence, the multi-layer network provides insights into the pattern of collective actions at the system/system-of-systems level.

The proposed framework conceptualizes the inter-organizational coordination among IISs embedded in urban systems as a multi-layer network. Each layer represents a specific infrastructure system (e.g., community development, transportation, flood control, emergency response, and environmental conservation) and nodes represent organizations. In terms of hazard mitigation in resilience planning, inter-layer links represent inter-organizational coordination within systems and intra-layer links represent coordination across systems. The simulation process perturbs inter-layer and intra-layer links based on their coordination probabilities (determined based on varying levels of collaboration frequency) within and across IISs. Accordingly, the inter-layer and intra-layer coordination level are determined using two measures related to network global efficiency. The details related to different steps of the proposed framework are discussed in the following section using a case study of Houston area, Texas, prior to Hurricane Harvey.

## Data

The city of Houston, Texas, is used as our study site to show application of the proposed framework. We conducted a survey of stakeholders in Harris County, Texas, to collect data regarding collaboration of hazard mitigation in resilience planning among organizations in different infrastructure systems (the survey was approved by Texas A&M University Human Subjects Protection Program office, and the written consent was obtained). After Hurricane Harvey in 2017, we identified 95 relevant organizations from five different infrastructure systems: transportation, flood control, emergency response, community development, and environmental conservation. We included these organizations in the survey as a roster of potential organizations with which survey respondents collaborated before Hurricane Harvey. We asked survey respondents about the frequency of hazard mitigation collaboration in resilience planning that occurred prior to Hurricane Harvey. We established the collaborative relationship through following survey questions: “*In the months or years prior to Hurricane Harvey*, *to the best of your knowledge*, *did you or any other employee from your organization collaborate or work directly with any of the organizations listed below on hazard mitigation efforts*? *If so*, *how frequently have such collaboration occurred (e*.*g*., *yearly*, *monthly*, *weekly and daily)*?*”* The survey was sent to stakeholders in February 2018 and concluded with a total of 198 individual responses representing 160 distinctive organizational departments.

**Fig 2:**
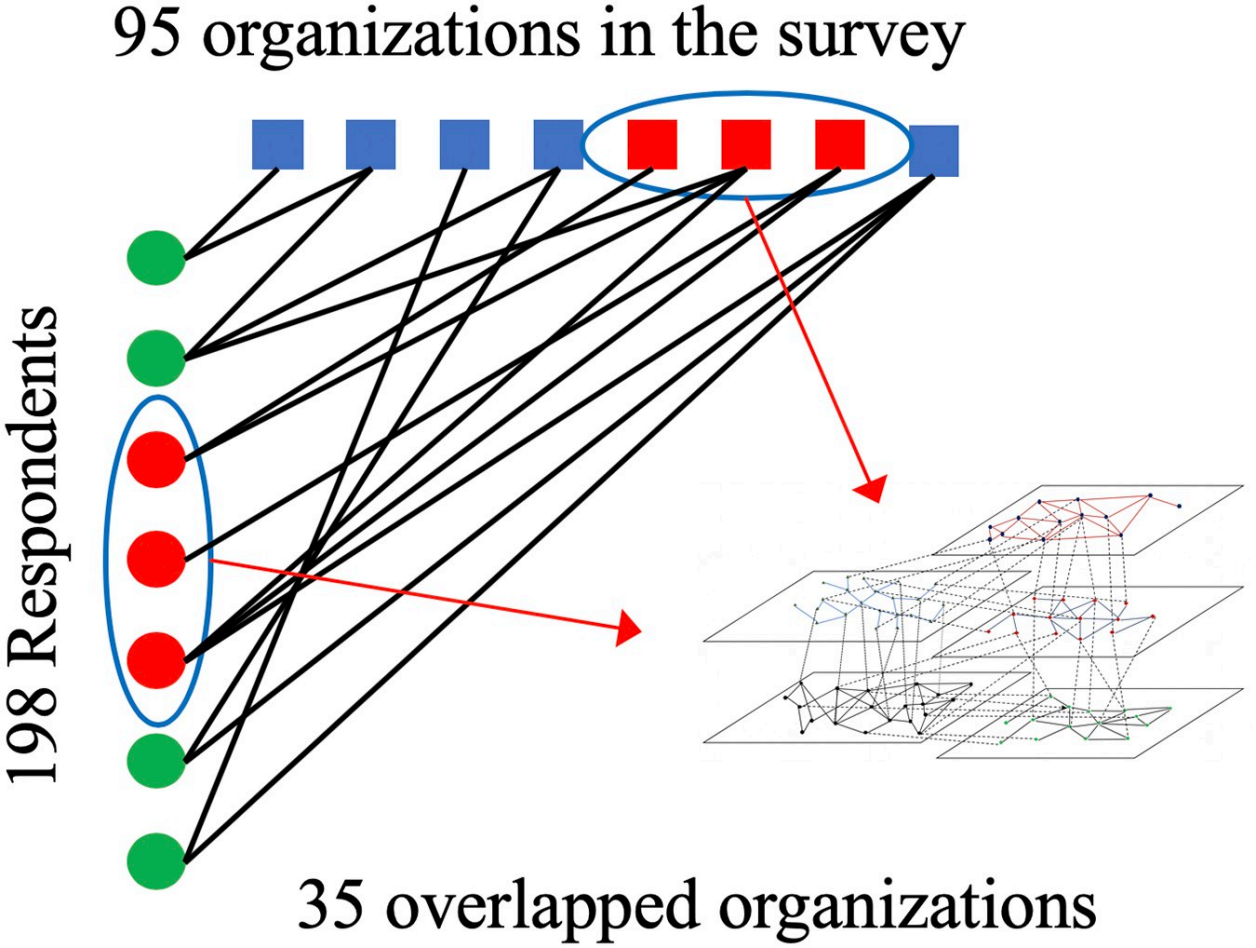
The collaboration network of 35 organizations.

Based on the gathered information regarding collaboration of hazard mitigation in resilience planning between organizations, we mapped two-mode (i.e., bipartite network) collaboration networks at different levels of collaboration frequencies (e.g., yearly, monthly, weekly and daily). Due to the nature of survey questions, relationships between organizations within the original survey roster and among the survey respondent organizations could not be determined. In consideration of this, we selected 35 organizations which were both in the survey roster and among the survey respondents to map the collaboration network. Fig 2 illustrates the process of mapping the collaboration network of these 35 organizations.

To understand inter-organizational coordination within and across different infrastructure systems, we categorized these 35 organizations into five infrastructure systems: flood control, emergency response, transportation, community development, and environmental conservation. Table 1 shows examples of organizations in each infrastructure system. Each infrastructure system is mapped into one layer of the multi-layer network model; links between organizations indicate coordination in terms of hazard mitigation. We then applied the simulation process to examine inter-organizational coordination among these 35 organizations prior to Hurricane Harvey.

**Table 1:** Examples of organizations in each infrastructure system.

Infrastructure system	Examples of organizations
Flood control	The Texas Floodplain Management Association, Texas Water Development Board, Harris County Flood Control District, Texas Coastal Watershed Program
Transportation	Metro, Texas Department of Transportation, Houston Transtar, Port of Houston Authority
Emergency response	Harris County Office of Emergency Management, Texas Department of Public Safety, Harris County Office of Emergency Management,
Environmental conservation	Bayou Land Conservancy, Bayou Preservation Association, Houston Wilderness, Urban Land Institute
Community development	Houston Real Estate Council, United Way of Greater Houston, Harris County Community Economic Development Department, West Houston Association

## Multi-layer simulation framework

The proposed framework comprises four main steps: (1) conceptualize inter-organizational coordination among IISs as a multilayer network; (2) determine coordination probabilities between organizations based on the reported frequency; (3) perturb links based on assigned coordination probabilities; and (4) evaluate the network performance after link perturbation using measures such as network efficiency and coefficient of variation. These steps are elaborated in the remainder of this section.

### Conceptualize inter-organizational coordination among IISs as a multilayer network

**Fig 3:**
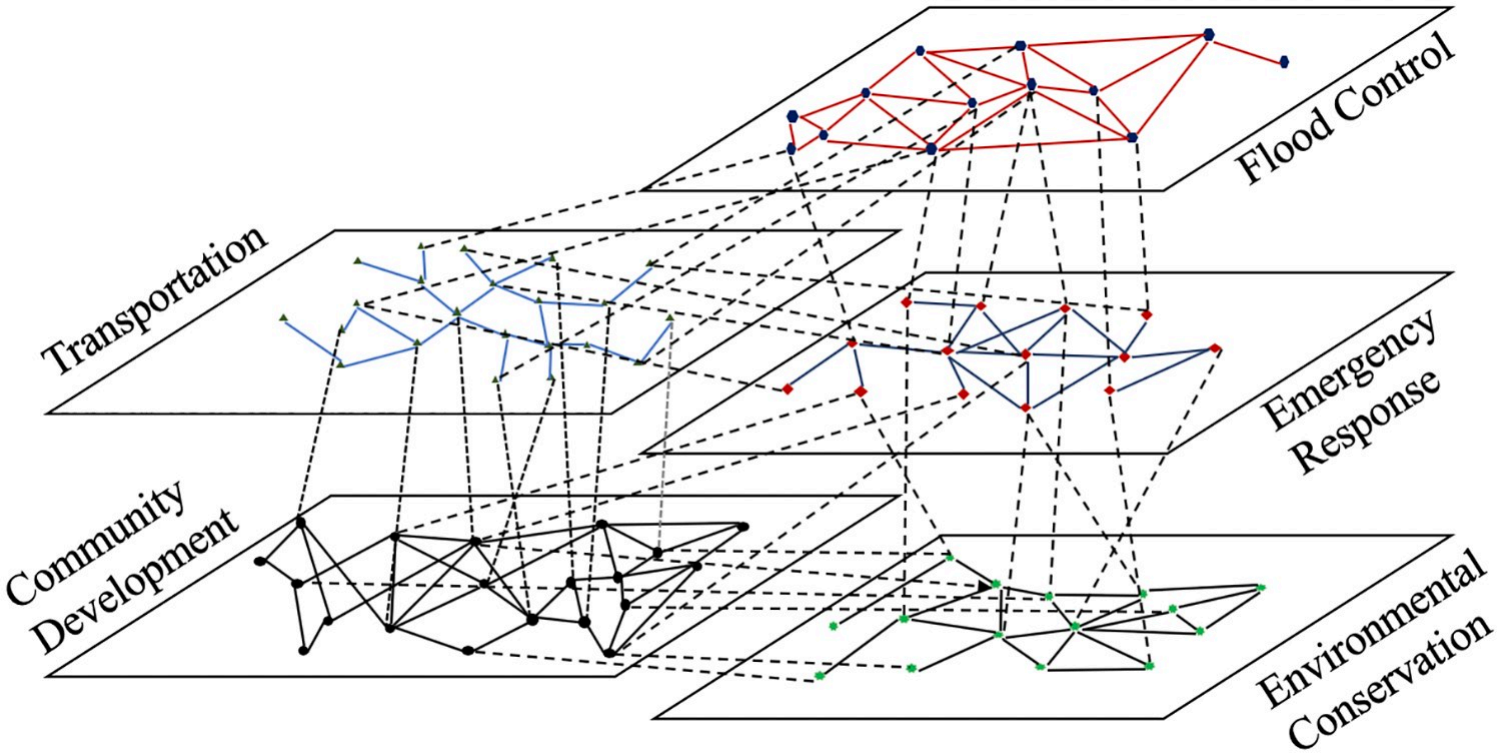
A multilayer network of five interdependent infrastructure systems.

The proposed framework conceptualizes coordination among organizations from different infrastructure systems as a multi-layer network. Each layer in the multi-layer network represents one infrastructure system. Intra-layer and inter-layer links of the multilayer network represent inter-organizational coordination for hazard mitigation in resilience planning within and across IISs. Fig 3 illustrates an example of a multi-layer network of five interdependent infrastructure systems.

### Determine coordination probabilities between organizations

Network simulations requires the probability of node (e.g., organization) or link (e.g., coordination) perturbation as input. But these mathematical probabilities are usually difficult to obtain directly. For example, coordination between organizations is often stated in frequency terms such as daily or weekly. Therefore, we use probability distribution to convert different levels of collaboration frequency (e.g., daily, weekly, monthly, and yearly) to the coordination probability of each link. We are able to obtain these levels of collaboration frequency from survey responses.

In the proposed framework, to determine coordination probabilities, we define daily interaction among organizations as the baseline, in which the probability of coordination between two organizations would be equal to one. We make the following assumptions to determine the daily coordination probability for other levels of collaboration frequency.

1. We approximate the probability distribution of coordination frequency as a normal distribution;
2. We define the boundaries for each frequency level (i.e., weekly, monthly, and yearly). The boundary for weekly collaboration is from once a week to seven times a week (e.g., 48–288 days per year) considering seven days one week. The boundary for monthly collaboration is from once a month to four times a month (e.g., 12–47 days per year) considering that one month has 4 weeks. Likewise, considering that one year has 12 months, the boundary for the yearly collaboration is from once a year to 11 times a year (e.g., 1–11 days per year). Finally, we consider daily frequency should have interaction at least once a day (e.g., ≥365 days per year). The boundaries of each collaboration frequency are listed in Table 2.
3. We treat holidays and weekends with the same weight as other days when determining coordination probabilities as they will not affect the simulation result, but unnecessarily, complicate the process.

**Table 2:** Converted daily coordination probabilities between organizations.

Collaboration frequency	Boundary (days per year)	Coordination probability
Daily	≥365	*P* = 1
Weekly	[48, 288]	$P\sim N(\frac{96}{365},\frac{24}{365})$ ^a^
Monthly	[12, 47]	$P\sim N(\frac{24}{365},\frac{6}{365})$ ^a^
Yearly	[1, 11]	$P\sim N(\frac{4}{365},\frac{1}{365})$ ^a^

^a^*N*(*μ*, *σ*) represents the normal distribution with mean *μ* and standard deviation *σ*.

Based on these assumptions, the daily coordination probability for organizations which reported a weekly interaction frequency is determined as an average of 96 days per year (i.e., twice a week) with a 95% confidence that the coordination frequency is in the interval of [48, 144] (i.e., once a week to three times a week). Likewise, the monthly coordination frequency is determined as 24 days per year (i.e., twice a month) on average with a 95% confidence to fall in the range of [12, 36] (i.e., once a month to three times a month). The yearly coordination frequency is determined as four days per year on average with a 95% confidence to fall in the range of [2, 6] (e.g., twice a year to six times a year). Table 2 summarizes the converted daily coordination probabilities at different collaboration frequency levels.

**Fig 4:**
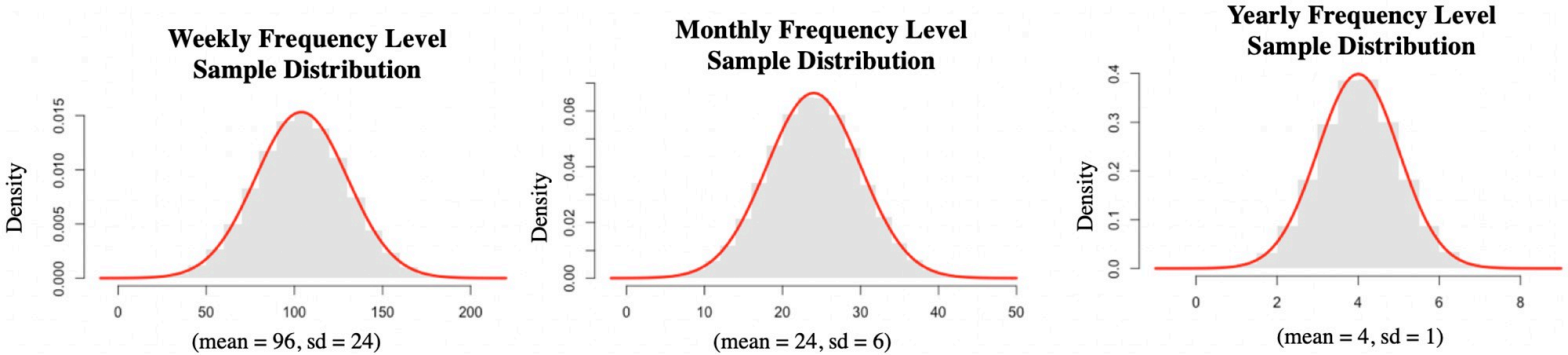
Histograms of generated samples at each frequency level.

Accordingly, the daily coordination probabilities are assigned to each link based on Table 2 for the network simulation process. Each normal distribution of weekly, monthly and yearly collaboration generates 100,000 samples during the simulation process. We compare the histograms of iterations for each normal distribution with their theoretical probability density functions. Fig 4 shows the histograms of samples at each collaboration frequency level. The results illustrate that 100,000 samples are large enough for the simulation process because the histograms of samples are very close to the theoretical probability density functions of each proposed normal distribution (indicated by the red curve in Fig 4).

### Perturb links based on assigned coordination probabilities

Each iteration of the simulation process would remove intra-layer and inter-layer links of the multilayer network based on the converted daily coordination probabilities (i.e., probability of perturbation is equal to 1—probability of daily coordination) between the organizations. First, we generate a random probability between 0 and 1 (0 and 1 themselves are excluded) in each iteration of the simulation process. Meanwhile, each link will randomly draw a sample among 100,000 generated samples for the assigned distribution. If the selected sample is less than the generated probability, the link is removed in this iteration of the simulation process. This probabilistic perturbation process means that the lower the daily coordination probability of the link is, the higher probability the link will be removed in the simulation process. For example, if the daily coordination probability of a link is 1 (i.e., coordination between organizations with daily frequency), the link would never be removed in the simulation process. We conduct the simulation process with 365 iterations to capture the inter-organization coordination fluctuation in a full year cycle. In the case of investigating the inter-organizational coordination within a specified infrastructure system, we only remove the correspondent intra-layer links. Accordingly, we only remove inter-layer links when examining the inter-organizational coordination across infrastructure systems. That means, if we want to investigate the inter-organizational coordination within the flood control system, we only remove the links within the flood control system (i.e., intra-layer links); when we want to analyze the inter-organizational coordination across the flood control and transportation systems, we only remove the links between these two systems (i.e., inter-layer links) in the simulation process. This separate link removal enables examining the level of coordination within and across different systems separately.

### Evaluate network performance after link removal

We adopt two measures for examining the level of coordination within and across IISs: network efficiency and its coefficient of variation after the link perturbation. Network efficiency measures the shortest distances between nodes after the link perturbation. The shortest distances between nodes tend to increase as links are removed, and the increase of distances between nodes can be interpreted as the decrease in the overall level of coordination among various organizations of IISs [36–38]. Network efficiency can be calculated using Eq 1 [39]:

$$E=\frac{1}{N(N-1)}\sum_{i,j}\frac{1}{d_{ij}}$$ (1)

Where *N* represents the total number of nodes in the network and *d*_*ij*_ is the distance of the shortest path between node *i* and *j*. Network efficiency is very sensitive to the total number of nodes (i.e., network size) in the network [40]. As a result, networks with great differences in size should not be compared by network efficiency. The coefficient of the network efficiency variation in multiple iterations can be calculated by Eq 2.

$$CV=\frac{\sigma }{\mu }$$ (2)

Where *σ* and *μ* are the standard deviation and mean of the network efficiency of multiple iterations. This measure implies the stability of coordination during the simulation process.

In order to evaluate the extent to which variation in daily coordination probability between organizations affects overall level of coordination, we examined five scenarios in which we uniformly assigned five different daily coordination probabilities (i.e., 15%, 30%, 45%, 60%, and 75%) to each link in the mapped multi-layer network.

### Examine coordination increase strategies

**Fig 5:**
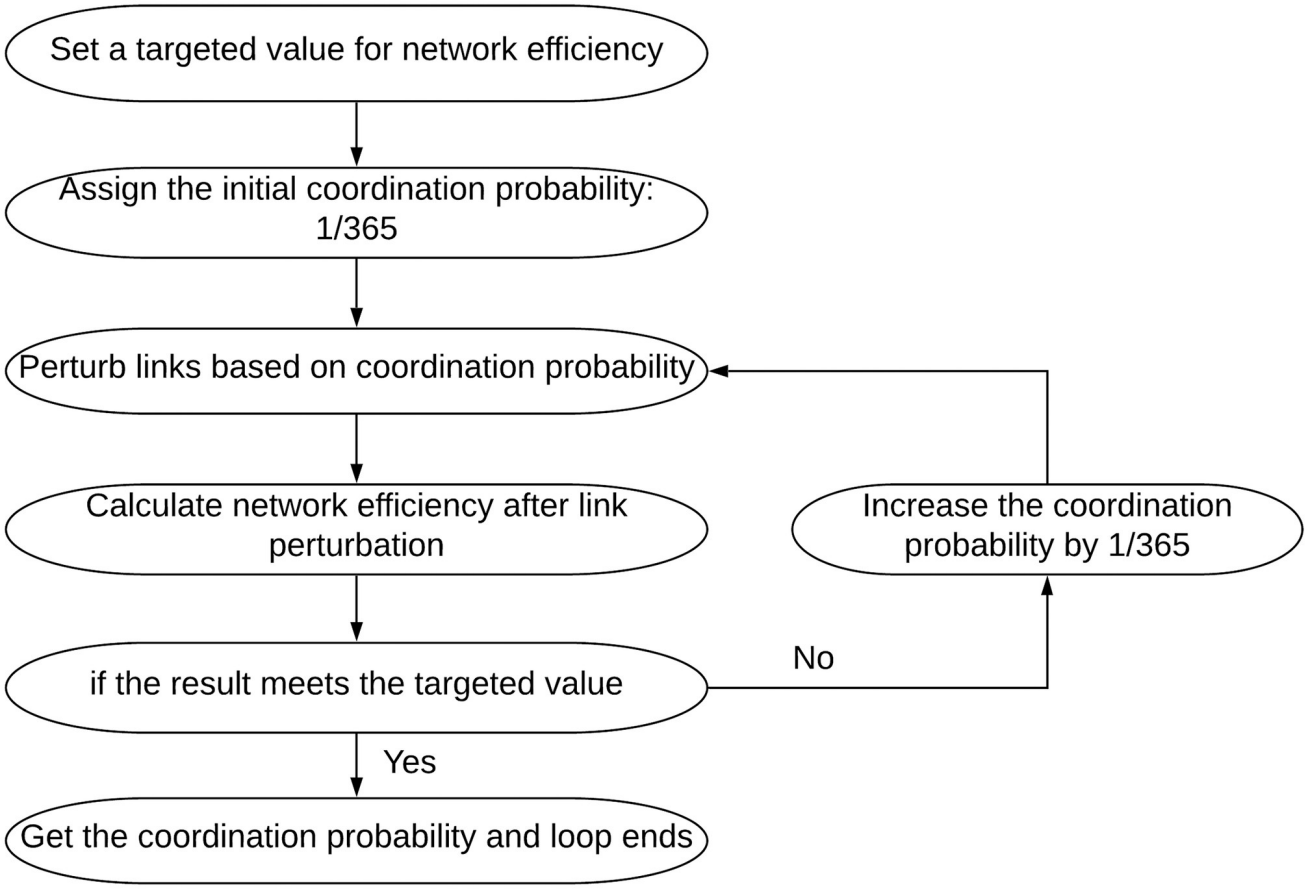
Coordination increase simulation to calculate required coordination probability.

To examine how to increase coordination within and across different systems, we conducted simulation experiments to determine the required coordination probability of links to achieve targeted network efficiency within and across different infrastructure systems. The first step is to uniformly assign each link an initial coordination probability: 1/365. Then the simulation process would be applied within or across different infrastructure systems, and network efficiency is calculated after simulation iterations. If the targeted network efficiency is not met, the coordination probabilities of links are increased by 1/365 increment until the targeted network efficient is met. Fig 5 shows the iterative mechanism to determine the required coordination probability for targeted network efficiency.

## Results

**Fig 6:**
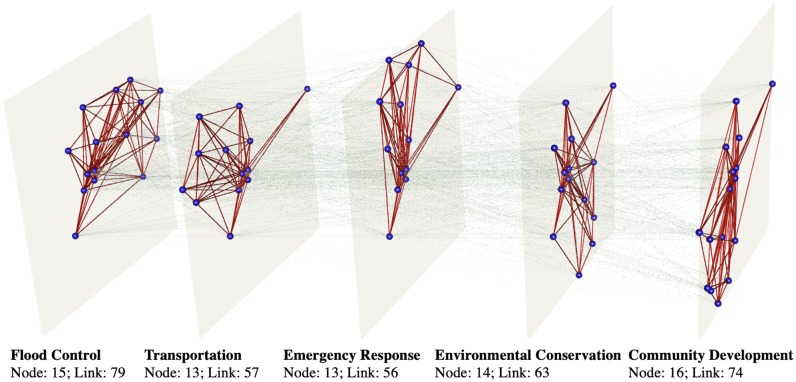
The multilayer collaboration network of 35 organizations.

In this section, we show the application of the proposed framework to the data collected from the stakeholder survey in Harris County, Texas. Each infrastructure system is mapped to one layer of the multi-layer network. Fig 6 illustrates the mapped multilayer network structure (generated by the software MuxViz [41]). The layers of the mapped multilayer network are all of similar size (Table 3) (e.g., the number of nodes in each layer is around 15).

**Table 3:** Nodes and links in each layer of the mapped meta-network.

Layer of mapped network	Nodes	Links
Flood control	15	79
Transportation	13	57
Emergency response	13	56
Environmental conservation	14	63
Community development	16	74
Total	71	329

The layer of flood control system has 15 nodes (i.e., organizations) and 79 links representing coordination between organizations in terms of hazard mitigation. The transportation layer comprises 13 nodes and 57 links, while the community development layer has 16 nodes and 74 links. The emergency response layer and environmental conservation layer have 13 nodes with 56 links and 14 nodes with 63 links, respectively. The total number of nodes in the five infrastructure systems is 71, which is more than the total number of organizations, 35. This is because some organizations, such as City of Houston, American Planning Association, and-Galveston Area Council, have multiple departments involved in different infrastructure systems, and therefore appear in more than one layer.

**Fig 7:**
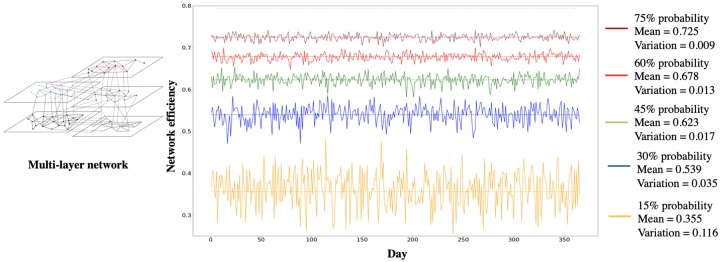
Network efficiency and coefficient of variation with different daily coordination probabilites.

The simulation results indicate that the lowest daily coordination probability for each link, 15% in this case, leads to the lowest mean of network efficiency (0.355) and the highest coefficient of variance (0.116). The scenario with the highest daily coordination probability for each link, 75%, leads to the highest mean of network efficiency (0.725) and the lowest coefficient of variance (0.009). Fig 7 illustrates that the higher daily coordination probability is (i.e., from 15% to 75%), the higher mean of network efficiency (i.e., from 0.355 to 0.725) and the lower coefficient of variance (i.e., from 0.116 to 0.009) will be after the simulation process. In the next step, we used the daily coordination probability based on the reported collaboration frequency between organizations from the survey. The simulation process perturbs intra-layer and inter-layer links based on the daily coordination probability (from the survey) in each iteration. Mean network efficiency and its coefficient of variation for intra-layer and inter-layer perturbation scenarios is illustrated in Tables 4 and 6. To juxtapose the network efficiency using the frequencies obtained in the survey with the maximum network efficiency, Tables 5 and 7 list the results of maximum network efficiency within and across different infrastructure systems. Maximum network efficiency is the greatest possible theoretical level of coordination among organizations and is determined when the daily coordination probability for all links in the network equals 1. In other words, maximum network efficiency is determined only by the network structure (e.g., the coordination among organizations of IISs) and will not be affected by the collaboration frequency.

**Table 4:** Network efficiency under intra-layer link perturbation.

Infrastructure system	Mean of network efficiency	Coefficient of variation
Flood Control	0.37	0.17
Transportation	0.46	0.13
Emergency Response	0.37	0.18
Community Development	0.25	0.23
Environmental Conservation	0.26	0.23

**Table 5:** Maximum network efficiency within IISs.

Infrastructure system	Maximum network efficiency
Flood Control	0.876
Transportation	0.865
Emergency Response	0.859
Community Development	0.808
Environmental Conservation	0.846

**Table 6:** Network efficiency under inter-layer link perturbation.

Infrastructure system	Mean of network efficiency	Coefficient of variation
Flood Control and Community Development	0.05	0.36
Transportation and Flood Control	0.17	0.40
Transportation and Community Development	0.01	1.73
Environmental Conservation and Flood Control	0.03	0.93
Emergency Response and Flood Control	0.16	0.33
Emergency Response and Transportation	0.28	0.20

**Table 7:** Maximum network efficiency across IISs.

Infrastructure system	Maximum network efficiency
Flood Control and Community Development	0.560
Transportation and Flood Control	0.652
Transportation and Community Development	0.667
Environmental Conservation and Flood Control	0.659
Emergency Response and Flood Control	0.707
Emergency Response and Transportation	0.762

As shown in Table 4, the transportation system has the highest mean value of network efficiency (0.46) and the lowest coefficient of variation (0.13). Community development and environmental conservation systems have the lowest mean of network efficiency (0.25 and 0.26, respectively) and the greatest coefficient of variation (0.23). This result indicates that the coordination within the transportation system is at a high level and consistent. On the other hand, coordination within community development and environmental conservation systems are at lower levels and more unstable.

As illustrated in Table 5, maximum network efficiency within different infrastructure systems is close, ranging from 0.808 (the community development system) to 0.876 (the flood control system). The comparison between the existing (pre-Harvey) network efficiency and the correspondent maximum network efficiency shows that, even for the transportation system (the highest within-system network efficiency), only about 50% of the maximum possible coordination is achieved. For the community development and environmental conservation, this value is approximately 30%.

Table 6 illustrates that, overall, the mean of network efficiency (0.12) across infrastructure systems is much lower and the mean of the coefficient of variation (0.66) is greater than those within infrastructure systems (i.e., 0.34 and 0.19, respectively). This implies a great number of missing and inconsistent cross-system coordination for hazard mitigation in resilience planning. Transportation and emergency response systems have the highest mean of cross-system network efficiency 0.28 and the lowest coefficient of variation 0.2. Transportation and community development systems show the lowest mean of cross-system network efficiency 0.01 and the highest coefficient of variation 1.73. The mean of cross-system network efficiency between flood control and community development systems is also low (0.05) (almost one-third) compared to the value between transportation and flood control systems (0.17). The results indicate that transportation and emergency response systems have a high level of cross-system coordination (i.e., more coordination and consistent). On the other hand, organizations in transportation and community development systems have a low level of cross-system coordination (i.e., less coordination and inconsistent). This is also true for the coordination across flood control and community development systems.

Table 7 indicates that maximum network efficiency across infrastructure systems is lower than the one within the systems, which can be another piece of evidence of missing cross-system coordination. Maximum network efficiency across community development and flood control systems is the lowest: 0.560; emergency response and transportation systems has the highest maximum network efficiency across the systems: 0.762. The comparison between the existing (pre-Harvey) network efficiency and the correspondent maximum network efficiency shows that, even for the highest network efficiency across transportation and emergency systems, only about 37% of the maximum possible coordination is achieved. For the coordination across transportation and community development systems, this value is nearly 1.5%.

**Fig 8:**
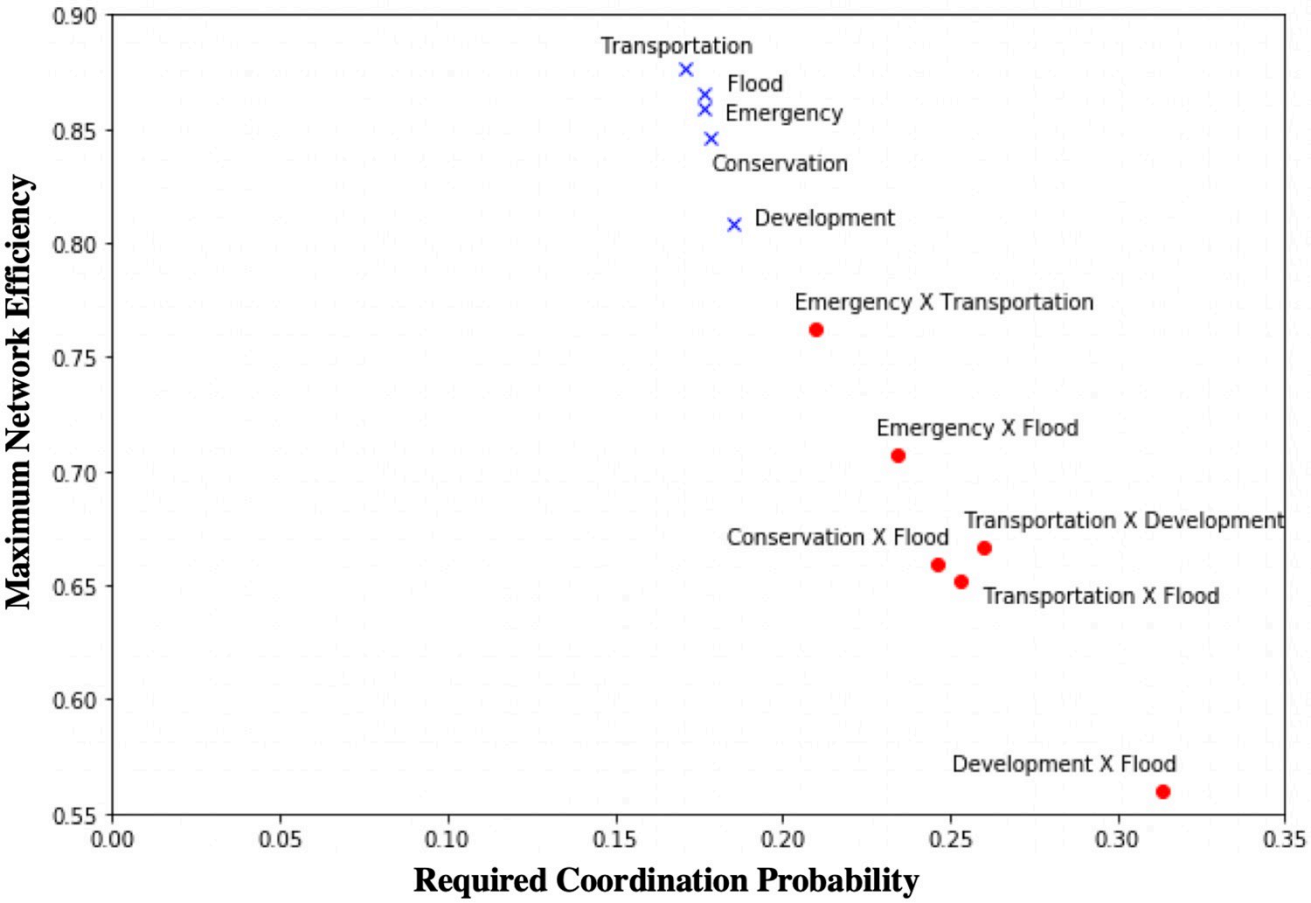
Required coordination probability and maximum network efficiency.

To study how to increase inter-organizational coordination within and across different infrastructure systems, we set the targeted network efficiency as half of the maximum values and applied the coordination increase simulation to calculate the required coordination probability. Tables 8 and 9 summarize the required coordination probability of links within and across IISs, respectively. Fig 8 illustrates the required coordination probability and maximal network efficiency.

**Table 8:** Required coordination probability within infrastructure systems.

Infrastructure system	Required coordination probability	Targeted network efficiency
Flood Control	*P* = 62/365 (weekly)	0.438
Transportation	*P* = 64/365 (weekly)	0.432
Emergency Response	*P* = 65/365 (weekly)	0.430
Community Development	*P* = 68/365 (weekly)	0.404
Environmental Conservation	*P* = 65/365 (weekly)	0.423

**Table 9:** Required coordination probability across infrastructure systems.

Infrastructure system	Required coordination probability	Targeted network efficiency
Flood Control and Community Development	*P* = 114/365 (weekly)	0.280
Transportation and Flood Control	*P* = 92/365 (weekly)	0.326
Transportation and Community Development	*P* = 95/365 (weekly)	0.334
Environmental Conservation and Flood Control	*P* = 90/365 (weekly)	0.330
Emergency Response and Flood Control	*P* = 85/365 (weekly)	0.354
Emergency Response and Transportation	*P* = 77/365 (weekly)	0.381

Tables 8 and 9 indicate that both required coordination probabilities within and across IISs are in the interval of weekly collaboration frequency level (nearly twice and three times a week, respectively). Although the targeted network efficiency within infrastructure systems are greater than those of cross-system values (due to their higher maximum network efficiency), the required coordination probabilities within infrastructure systems (averaging 65/365) are lower than those across systems (averaging 92/365). This implies that achieving a high level of coordination across different systems is more difficult and would require greater frequency of interactions (compared to within system coordination). The flood control system has the highest targeted network efficiency: 0.438 while having the lowest required coordination probability: 62/365. Meanwhile, coordination across flood control and community development systems has the lowest targeted network efficiency: 0.280, while it needs the greatest coordination probability: 114/365. This finding implies that the flood control system can achieve a higher level of coordination more easily compared to other infrastructure systems due to its better collaboration network within the system. However, the interaction between flood control and community development systems is lower, and it would be more difficult to achieve a higher level of cross-system coordination.

Fig 8 illustrates that compared to cross-system coordination, within-system coordination can achieve greater network efficiency with relatively lower frequencies of interactions. Cross-system coordination, however, could achieve a lower maximum network efficiency and requires higher frequencies of interactions among organizations across systems. This would imply that a high level of cross-system coordination is harder to achieve based on the current collaboration network and would require a greater frequency of cross-system interaction and perhaps different mechanisms (e.g., add more links/establish new collaboration) for achieving a high level of cross-system coordination.

## Discussion

The results of the multi-layer network simulation framework examine inter-organizational coordination in IISs for flood hazard mitigation in Harris County. The discussion of the results below focuses on how the level of inter-organizational coordination within and across IISs may affect resilience planning outcomes. We provide anecdotal evidence and link to other studies to reinforce the validity of the findings obtained from the multilayer network simulation framework.

### Inter-organizational coordination within the infrastructure systems

As shown by the results, the maximum network efficiency within different infrastructure systems are close (around 0.85), which implies that different infrastructure systems have almost the same potential to reach the same level of within-system coordination. However, the actual coordination for each system varies based on the collaboration frequency obtained from the survey, with the highest level of coordination (transportation: 0.46) being almost twice that of the lowest (community development: 0.25). The transportation system has the highest network efficiency and the lowest coefficient of variance after the intra-link perturbation, implying that prior to Harvey, organizations within the transportation system had a higher level of coordination with each other compared to organizations in other infrastructure systems.

The flood control and emergency response systems have the second highest network efficiency and the second lowest coefficient of variance for within-system coordination. Also, the flood control system has the highest maximum network efficiency. The flood control and emergency response systems also had relatively high levels of coordination for hazard mitigation prior to Harvey. The flood control system plays an important role in hazard mitigation and resilience planning. Organizations such as Harris County Flood Control District and Texas Floodplain Management Association within the system are usually responsible for floodplain management and hazard mitigation plan and policy development (e.g., flood control plan and policy of building foundation level lift). Sufficient inter-organizational coordination within the flood control system would be an important foundation for hazard mitigation integration in resilience planning [5]. Organizations from the emergency response system, such as Harris County Office of Emergency Management and Texas Department of Public Safety, play an important role in response and recovery during and after a disaster [17,42]. Other studies [43–45] have also shown that enhanced coordination between organizations within the emergency response system would greatly improve emergency response processes, and thus community resilience.

The network efficiency within the community development system is the least, and it also has the lowest maximum network efficiency. One possible reason is that there is a lower level of coordination among organizations within the community development system compared to other infrastructure systems in Harris County. This finding suggests that organizations within the community development system do not fully engage in hazard mitigation processes. Coordination for hazard mitigation among organizations in the community development system is crucial for resilience planning [3,10]. However, as survey data and results show, adequate coordination within the community development system was missing prior to Harvey.

### Inter-organizational coordination across different infrastructure systems

The simulation results show that the level of coordination across different infrastructure systems is much lower than the coordination level within infrastructure systems. Also, the maximum network efficiency across systems is lower than those within the infrastructure systems. This means that inter-organizational coordination for hazard mitigation across infrastructure systems is harder to achieve a high level compared to within-system coordination. The highest level of coordination within the system (transportation: 0.46) is nearly 1.6 times more than the highest one across systems (transportation and emergency response: 0.28). Organizations in the emergency response system have a high level of coordination for hazard mitigation with organizations in the transportation system indicated by the highest cross-system network efficiency and lowest coefficient of variance. This could be due to the importance of transportation infrastructure (e.g., highways and bridges) in emergency response operations (such as evacuation and relief supply). For example, many roads in Houston were flooded with water more than 25 inches deep during hurricane Harvey, preventing access by fire vehicles. Firefighters had to manage rescues by boat, greatly decreasing rescue efficiency.

The results also show that organizations in the flood control system have a low level of coordination with organizations in the community development system. Also, inter-organizational coordination for hazard mitigation between community development and transportation systems is the lowest despite the high maximum network efficiency. This finding suggests that organizations from the community development system had insufficient coordination for hazard mitigation with organizations in other infrastructure systems prior to Hurricane Harvey.

### Coordination increase strategies: Establish new collaboration or increase frequency

The results indicate two ways to increase the level of inter-organizational coordination within and across systems: (1) increasing maximum network efficiency, and (2) increasing coordination probabilities. In the context of resilience planning, these two methods essentially suggest establishing new collaborations or increasing coordination frequency among organizations. Maximum network efficiency relates to the number of the shortest paths in networks. Basically, for the same network, a network of greater density implies higher maximum network efficiency [39]. This implies that adding links in the network, especially links with higher betweenness centrality nodes, will greatly increase the maximum network efficiency [39]. On the other hand, for the same network, when the maximum network efficiency is relatively high, increasing maximum network efficiency by adding new links would be difficult because it requires large numbers of new links (the density of the network is proportional to *n*^*2*^, where *n* is the number of nodes in the network). However, when the maximum network efficiency is low, it requires great coordination frequency to increase the network efficiency.

In summary, to increase the level of inter-organizational coordination within and across IISs, the findings suggest establishing new interactions among organizations when the existing collaboration is small and limited, especially with organizations involved in more than one infrastructure system (such as City of Houston, American Planning Association, and Houston-Galveston Area Council). Forums and workshops in which diverse actors could participate are considered an effective way to establish new interaction regarding resilience planning [46]. Also, organizations at higher administration levels (such as City of Houston, and Texas Department of Transportation) could play a boundary-spanning role to help establish coordination among organizations at lower administration levels (such as Houston Transtar and Houston-Galveston Area Council) [47]. Furthermore, the combination of establishing new collaboration and increasing interaction frequency would also be a good strategy. Organizations in IISs could establish new collaboration with the aforementioned organizations involved in multiple infrastructure systems or at higher administration levels and increase the interaction frequency with the ones with which they already have established coordination.

Although the planning background in Houston and the example of Hurricane Harvey suggest that more coordination among IISs would have enhanced outcomes in resilience planning, it is important to note that a body of literature highlights that there are often tradeoffs and unintended consequences of higher connectivity and coordination in networks [48–53]. Chelleri et al. studied interactions across scales and systems resulting in resilience trade-offs, and one case study showed that greater community cohesion does not necessarily lead to greater community resilience [49]. Chen et al. found that increased internal interactions in the large-scale infrastructure systems composed of many shared public facilities may lead to greater vulnerability and large-scale failures [50]. Shutters et al. studied the relationship between system connectedness and resilience. The results showed that in response to a shock, cities with lower social-economic system connectedness have higher resilience [51]. Ulanowicz et al. found that tightly constrained ecosystems appear ‘brittle’ to disruptions [52]. Panarchy theory pointed out that social-ecological systems with strong interdependencies may have lower resilience as one node failure may lead to cascade failures in the system [53]. While greater connectivity is shown to be correlated with a greater system vulnerability in some physical and ecological systems, some studies showed that this could be generalized to human systems as well. Burt argued that a highly dense network would lead to redundant connection and decrease the efficiency of communications among actors [54]. In another study, Burt and Granovetter showed that social capital lies in the weak ties between structural holes in human networks [48,55]. Based on these findings, it could be the case that increasing coordination among diverse actors may not necessarily lead to better outcomes in resilience planning. Nevertheless, in this paper, we primarily focus on examining inter-organizational coordination and understanding how organizations may increase coordination within and across IISs.

## Concluding remarks

This paper proposes and tests a multilayer framework for simulating the network dynamics of inter-organizational coordination among IISs in resilience planning. The proposed framework and its application in the context of Harris County, Texas, prior to Hurricane Harvey have multiple methodological and theoretical contributions. First, the presented work considers the organizational aspects of interdependencies among IISs, departing from the majority of infrastructure interdependency studies which mainly focus on physical aspects. Second, the proposed framework adopts the simulation process and multilayer network for examining human/organizational networks with heterogenous types of nodes and dynamic links. The proposed framework enables modeling inter-organizational coordination among IISs with heterogenous nodes and capturing the coordination frequency among the nodes. Third, the framework provides new insights into coordination dynamics among organizations within and across different systems. Fourth, from the practical perspective, the framework enables examining the coordination increase strategies and provides recommendations to increase the level of coordination among organizations, which may lead to better resilience planning in IISs.

Some limitations in the proposed framework still exist which can be addressed in future research. This study assumes that coordination frequency is normally distributed. Future studies can further test this assumption by estimating the coordination probability based on the longitudinal data gathering regarding interaction frequencies. Such data collection would require more specific data regarding the dates and mode of coordination interactions among agencies, a task whose implementation would not be straightforward. Also, in the simulation experiments for examining the coordination increase strategies, we increased the same coordination probability for all links in the scenarios (1/365 in each iteration). The future work could examine the increase in coordination probability based on different network reticulation mechanisms, such as preferential attachment. Furthermore, inter-organization networks directly and indirectly influence the networks of plans, as well as infrastructure networks. A low level of coordination in inter-organizational networks may lead to conflicting plans and more vulnerability in physical networks. Hence, understanding the interdependencies among inter-organizational networks, network of plans, and physical networks may hold the key to unlocking a holistic resilience planning process in IISs.
